# Oral food challenges: practical aspects

**DOI:** 10.1186/2045-7022-1-S1-S18

**Published:** 2011-08-12

**Authors:** Odilija Rudzeviciene

**Affiliations:** 1 Vilnius University, Clinic of Children Diseases, Vilnius, Lithuania

## 

Aims of controlled oral food challenges are both to prove that a certain allergen plays a role for the individual clinical symptoms and to exclude food allergy in order to prevent the child from unnecessary or even harmful elimination diets. There are such types of challenges in the clinical practice: open, single-blind, or double-blind, placebo-controlled. Open OFC is an unmasked, unblinded feeding with a food in its natural form. However, it has the highest potential for bias, which may depend on age, personality, and type of symptoms. In the single-blind OFC, the observer but not the patient knows the food being tested. In the double-blind OFC, challenge material is provided by a third party, such as a dietitian, whereas the patient, the patient’s family, and the observer are unaware of when the test food is given. All suspected foods should be strictly avoided for a sufficient period before OFC. The elimination period will usually last 1-4 weeks depending on the symptomatology and should abolish or at least markedly reduce symptoms. OFC should always be performed under controlled professional supervision. Patients must be in good health, and their allergic diseases should be under optimal control at the time of the OFC. Discontinuation of medications that may interfere with the OFC may be needed. Usually it is recommended to obtain written informed consent from the parent or guardian for OFC. It is necessary to examine the patient and record all data in the challenge protocol. Also it is necessary to prepare rescue medication, calculate the dosage and record in the challenge protocol. Intravenous access should be available always if a severe reaction is expected. The patient should be re-examined before each dose is administered. In case of an allergic reaction to OFC, treatment should be initiated promptly.

